# Shifts in avian migration phenologies do not compensate for changes to conditions en route in spring and fall

**DOI:** 10.1002/ecy.70110

**Published:** 2025-05-18

**Authors:** Carrie Ann Adams, Monika A. Tomaszewska, Geoffrey M. Henebry, Kyle G. Horton

**Affiliations:** ^1^ Department of Fish, Wildlife, and Conservation Biology Colorado State University Fort Collins Colorado USA; ^2^ Center for Global Change and Earth Observations Michigan State University East Lansing Michigan USA; ^3^ Department of Geography, Environment, and Spatial Sciences Michigan State University East Lansing Michigan USA

**Keywords:** aeroecology, aves, birds, climate change, green‐up, land surface phenology, migration, phenology, radar ornithology

## Abstract

Several factors are known to affect bird migration timing, but no study has simultaneously compared the effects of temperature, land surface phenology, vegetation greenness, and relative humidity in both spring and fall. In addition, it is unclear whether long‐term shifts in migration phenologies have kept pace with changing climates. For example, if migration shifts earlier in the spring, temperatures on migration dates may remain stable over time despite spring warming trends. If the phenologies of birds, plants, and insects shift asynchronously in response to changing climates, then birds may encounter reduced resource availability during migration. We estimated spring and fall 10%, 50%, and 90% cumulative migratory passage dates at 53 weather surveillance radar stations across the US Central Flyway. We determined which conditions (temperature, timing of green‐up and dormancy, relative humidity, and enhanced vegetation index [EVI]) explained annual variation in migration phenologies. We also described decadal trends in environmental conditions and whether shifts in migration phenologies were sufficient to compensate for these changes. Annual changes to spring migration phenologies were best explained by anomalies in temperature, with earlier passage in warmer years. Fall migration occurred later on warmer, more humid years with higher EVI and later dormancy. Long‐term adjustments in bird migration phenologies did not mitigate their exposure to changing environmental conditions. Although passage dates for all spring migration quantiles advanced significantly (~0.6 days/decade), temperatures on spring 10% passage dates increased, while 50% and 90% passage occurred closer to green‐up. In the fall, temperatures increased on 50% and 90% passage dates. By contrast, the advancement of 10% passage (~1 day/decade) prevented early migrants from experiencing the cooling late‐summer temperature trend. Warmer temperatures in mid to late fall may lead to earlier fruiting phenology and asynchronies with migratory passage, which occurred later in warmer years. Changes in temperature and land surface phenophases experienced by migrants suggest that resource availability during migration has changed and that adjustments to migration phenologies have not compensated for the effects of changing climates.

## INTRODUCTION

### Background

Cumulative emissions of greenhouse gases have reshaped climate systems (IPCC et al., 2022). Global temperatures are rising, severe weather events are increasing, and spring events are occurring earlier in the year. Phenology, the timing of life history events, can shift in response to changing climates in different magnitudes or directions across trophic levels (Kharouba et al., 2018; Prather et al., 2023; Thackeray et al., 2016). Increasing phenological asynchronies can result in phenological mismatches between trophic levels when they negatively impact consumer fitness and population growth (Samplonius et al., 2021). Phenological asynchronies and resulting mismatches may be particularly likely for migratory species if changes on their breeding grounds are decoupled from changes in areas where they spend the non‐breeding season, and these mismatches can lead to population declines (Both et al., 2006; Jones & Cresswell, 2010; Youngflesh et al., 2023). Phenological asynchronies may be avoided or reduced if migration phenologies shift, through behavioral plasticity or selective pressures, to correspond with changes in resource phenologies.

Previous research on spring migration timing has focused on either green‐up or temperature, without comparing their effects or considering other environmental conditions, and has primarily measured conditions upon arrival at the breeding sites, not en route. Green‐up, the spring timing of new vegetation outside of the tropics (Schwartz & Marotz, 1986), is one aspect of land surface phenology (LSP) that can be remotely sensed to quantify seasonal patterns in vegetated land surfaces (Henebry & de Beurs, 2013). Temperature, land surface phenophases, relative humidity, and vegetation biomass can all affect the availability of insects for migratory birds en route and at breeding sites (Chown et al., 2011; Nash et al., 2023; Shipley et al., 2022; Williams, 1961; Winkler et al., 2013). Migrating birds in the continental United States have shown some interannual plasticity in arrival dates, arriving earlier in years with earlier green‐up (Youngflesh et al., 2021) or higher temperatures (Ellwood et al., 2010; Horton, Morris, et al., 2023; but see McDonough Mackenzie et al., 2019; Miller‐Rushing et al., 2008). Migration phenology en route from the non‐breeding to breeding areas is less well documented than arrival dates and has been found to occur earlier in years with higher temperatures (Horton, Morris, et al., 2023). In addition to the evident interannual plasticity, trends in resource phenologies may drive the evolution of earlier migration and breeding dates for migratory species (Charmantier & Gienapp, 2014; Marrot et al., 2018). Spring temperatures are rising, and vegetation phenologies may be occurring earlier over time (Moon et al., 2021; Piao et al., 2019), though analyses within the continental United States have found delaying trends, especially in agricultural landscapes (Liang et al., 2021; Zhang et al., 2019). Spring migratory passage and arrival dates in the United States are also advancing (Horton et al., 2020), though the trend is not universal across species or locations (Ellwood et al., 2010; Horton, Buler, et al., 2023).

Much less research has focused on the drivers and trends of fall phenologies, including bird migration (Gallinat et al., 2015). Some fall migrants migrate earlier in warmer years (Horton et al., 2020; Horton, Morris, et al., 2023), but potential effects of LSP on fall migration patterns have not been examined. Although several studies have reported a trend toward delayed LSP in the fall (e.g., Piao et al., 2019), these trends are spatially variable and are trending earlier in some parts of the Central Flyway (Liang et al., 2021). If fall LSP is delayed, there may be selective pressures on fall migration phenology, such as delayed migration due to increased opportunities for second broods (Both et al., 2019) or delays in fruit phenologies (Stiles, 1980; Thompson & Willson, 1979). Fruits are an important resource for fall migrants in stopover sites (Parrish, 1997, 2000). With warming temperatures and changes to LSP, there may be asynchronies between fall fruits and the birds that consume and disperse them (Gallinat et al., 2018, 2020). In the Central Flyway, peak migratory passage detected by radar occurred ~26 days before dormancy across all latitudes (Adams et al., 2024), which may indicate an important synchrony between the southward progression of vegetation phenophase and avian migration in the fall.

Shifting migration phenologies may not be sufficient to maintain long‐term stability in the conditions experienced by migrants en route. While several studies focus on rising temperatures and changes in arrival proximity to green‐up on the breeding grounds (e.g., Both et al., 2006; Mayor et al., 2017; Youngflesh et al., 2021), we do not know whether avian migrants are experiencing long‐term changes in a suite of environmental conditions en route, particularly in the fall. A recent study showed that spring migrants in North America are experiencing earlier LSP along migration routes as green‐up dates advance (Robertson et al., 2024), though this trend may be reversed in the Central Flyway if green‐ups are occurring later (Liang et al., 2021). As climates change, migratory bird populations that do not shift their migration phenologies may experience phenological mismatches and become more vulnerable to population declines (Jones & Cresswell, 2010; Kelly & Cimprich, 2024; Youngflesh et al., 2023). Studying the extent to which changes to migration phenologies have kept pace with changes in temperature, LSP, relative humidity, and vegetation greenness patterns in the fall as well as the spring is crucial to understanding migratory birds' ability to adapt as climates change.

**FIGURE 1:**
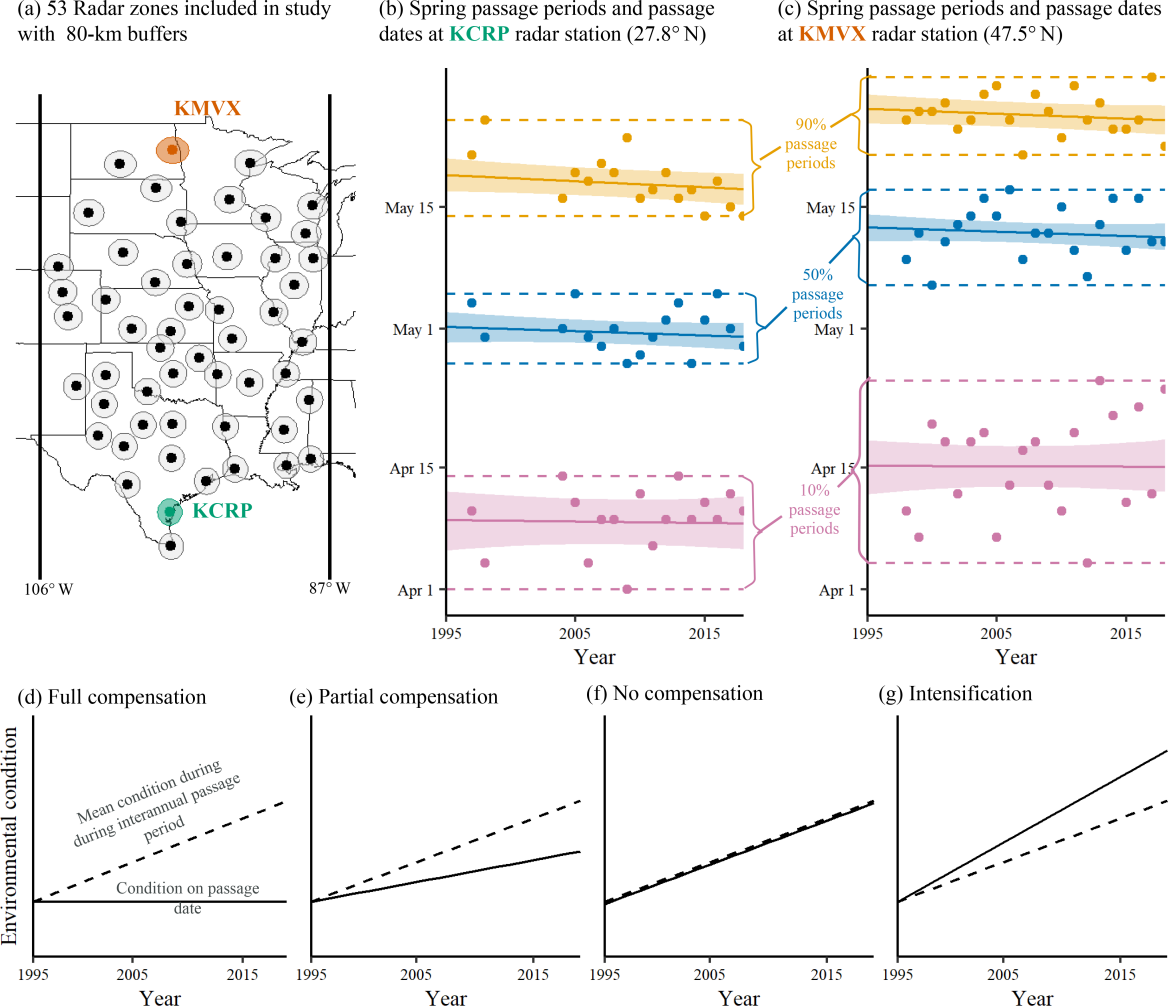
Study area map, definition of passage periods, and hypothetical scenarios for how changes to migration phenologies could compensate for environmental changes experienced by migrants. Panel (a) shows the study areas and radar zones. Panels (b/c) show two stations' passage periods (dashed lines), defined by the earliest and latest day‐of‐year that each passage quantile occurred at each station across all years. Points represent passage dates. Solid lines and shaded areas show trends in passage dates and 95% credible intervals (CIs). Panels (d–g) show four scenarios for how shifting migration phenologies could prevent the assemblage of migrants from experiencing changes in environmental conditions that occur during passage periods.

To study how interannual and long‐term trends in bird migration phenology relate to changing environmental conditions in both the spring and the fall, we used weather surveillance radar (WSR) to measure nocturnal migratory passage in the Central Flyway (Figure 1a). More than 70% of bird species in North America migrate, and over 80% of those species migrate at night (Horton et al., 2019). Nocturnal migrants include most waterbirds, shorebirds, and songbirds, while soaring species like raptors migrate during the day. We quantified migration phenology using the dates of 10% (early), 50% (peak), and 90% (late) cumulative passage and used these data to answer three research questions. There is substantial variation in bird species' phenological trends and responses to temperature (Horton, Morris, et al., 2023), which is obscured by dividing the assemblage of avian migrants into early, peak, and late migration cohorts, but WSR offers other advantages. Much of our knowledge about avian response to environmental changes originates from studies that are location‐specific (Ellwood et al., 2010; McDonough Mackenzie et al., 2019; Miller‐Rushing et al., 2008), taxa‐specific (Horton, Morris, et al., 2023), track a small number of individuals (Nemes et al. 2023), and/or focus on migrants arriving at their breeding locations (Youngflesh et al., 2021, 2023). Using WSR enabled us to capture continental movements of nocturnal migrants en route at an aggregate level and to sample across long time periods in both spring and fall.

### Objectives

Our first research question was “do birds change their annual migration phenology in accordance with annual changes in environmental conditions (temperature, LSP, relative humidity, and the enhanced vegetation index)?” We predicted that migration would occur earlier in the spring and later in the fall in years with higher temperatures, relative humidity, and enhanced vegetation index (EVI), as well as earlier green‐up in spring and later dormancy in fall. Such years are associated with earlier resource availability (Moon et al., 2021), higher insect abundance, and an extended growing season (Gill et al., 2015), allowing birds to migrate earlier and remain longer on breeding grounds or in fall stopover sites.

Second, we asked, “how much did bird migration phenologies in the Central Flyway change between 1995 and 2018?” In accordance with previous studies, we predicted that migration would advance in both spring and fall (Van Buskirk et al., 2009; Horton et al., 2020).

Our third question asked how shifts in migration phenology affected the conditions experienced en route. Specifically, “were the shifts in migration phenologies sufficient to compensate for any long‐term trends in environmental conditions such that conditions experienced by migrants remained consistent over time?” We compared the trends in environmental conditions on the observed cumulative passage dates (dates when 10%, 50%, and 90% of cumulative passage occurred in each radar zone/season/year combination) to the trends that occurred in each radar zone during the interval when each migration quantile typically passed (passage periods, Figure 1b/c). If migrants responded to environmental conditions by shifting their migration dates, we predicted that the conditions experienced by migrants would remain unchanged over time (full compensation, Figure 1d) or would change less than the overall trends in environmental conditions that occurred during the passage periods (partial compensation, Figure 1e). If migration phenologies did not shift enough to keep pace with trends in environmental conditions, we predicted that conditions experienced on passage dates would show the same trends as conditions during the passage periods (no compensation, Figure 1f). Migrating earlier or later could intensify trends in environmental conditions experienced by migrants (intensification, Figure 1g) if, for example, migration phenology shifted toward a warmer part of the passage period and temperatures warmed over time. Specifically, we predicted that spring migrants might not experience the full extent of increased temperatures or changes in passage relative to green‐up that occurred during their passage periods if they advanced their migration phenology over time and in warmer years with earlier green‐up (full or partial compensation). Because migration phenologies may be advancing in the fall (Horton et al., 2020), and conditions earlier in the season are generally warmer, drier, and farther from senescence, we predicted that trends in environmental conditions experienced by fall migrants could exceed trends observed during the passage periods (intensification).

## METHODS

### Data acquisition

#### Migratory passage phenology

We studied migration phenologies in spring and fall for the years 1995 through 2018 at 53 WSR stations within the US Central Flyway, only including stations with at least 10 years of data collected during this period (Figure 1a). The Central Flyway is the most active migratory flyway in the United States (Horton et al., 2019) and spans longitudes 87° W–106° W (La Sorte et al., 2014). Horton et al. (2020) describe in detail how phenology estimates were generated. In summary, we calculated reflectivity (in square meters per cubic kilometer), migrant direction, and flight speed within 100 km of each radar (hereafter called radar zones). These radar measures were aggregated into altitudinal bins between 100 and 3000 m above ground level, removing stationary clutter and precipitation prior to aggregation. We estimated nightly passage over each radar zone by multiplying the reflectivity by the northward (spring) or southward (fall) component of the measured ground speed (in kilometers per hour), summing through the air space and integrating through the night using linear interpolation for the area under the curve with the *auc* function in the *MESS* package (Ekstrøm, 2023), which uses a composite trapezoid rule. This process generated an estimate of the number of birds passing the radar zone per night, which we assembled across each season to generate curves of cumulative passage. We calculated the dates on which 10%, 50%, and 90% cumulative passage occurred for each radar zone in each year and season (hereafter called passage dates). Most birds detected were likely nocturnally migrating songbirds, particularly during the 50% passage dates (Dokter et al., 2018; Horton et al., 2019; Haas et al., 2021; Kelly et al., 2016; Nilsson et al., 2018).

#### Passage periods

To analyze trends in environmental conditions, we needed a consistent and biologically relevant period during which to compare environmental conditions across years. We defined passage periods for each quantile (10%, 50%, and 90%) as the interval between the earliest and latest day‐of‐year that each passage date occurred at each station (Figure 1a/b). Thus, these radar‐specific passage periods included the same dates across years but different dates for each radar and for each passage quantile.

#### Land surface variables within the Central Flyway

We estimated daily mean air temperatures and relative humidity at 2 m above ground level for 1995–2018 from the NCEP North American Regional Reanalysis (NARR) at a 32‐km spatial resolution (Mesinger et al., 2006). Vegetation biomass was estimated using the EVI from MODIS product MOD13A1.061 (Didan, 2021), using 16‐day composites at a 500‐m resolution. We used LSP date estimates from MCD12Q2.061 (Friedl et al., 2022) at a 500‐m resolution for 2003 (when MODIS on Aqua satellite first collected a full year of data) to 2018 for dates of two phenophase transitions: green‐up (date when EVI2 first crossed 15% of the EVI2 amplitude) and dormancy (date when EVI2 last crossed 15% of the EVI2 amplitude). We calculated the proximity of each passage date to the phenophase transition date by subtracting the passage date from the green‐up or dormancy date.

We spatially aggregated data for each environmental condition by calculating the mean of all pixels in an 80‐km radius around the radar station each year. For the analyses of long‐term trends in environmental conditions experienced on passage dates, we used these spatial means as the response variables. For the analyses of environmental conditions present during the migratory passage period, we temporally aggregated temperature, EVI, and relative humidity data by calculating the mean values within passage periods.

#### Climate oscillation modes

To account for the effects of climate oscillation modes on North American LSP (Dannenberg et al., 2018; McCabe et al., 2012) and bird migration phenology (González‐Prieto & Hobson, 2013; Zuckerberg et al., 2020), we included indices of the El Niño and Southern Oscillation (ENSO) and Northern Annular Mode (NAM) in our models. We downloaded values for The Multivariate ENSO Index Version 2 (MEIv2) from https://psl.noaa.gov/enso/mei, which lists MEIv2 values for partially overlapping two‐month periods. We averaged across the periods to obtain one value for winter (mean of November/December, December/January, January/February), spring (mean of March/April, April/May, May/June), and fall (mean of July/August, August/September, September/October) each year. We also determined the MEIv2 values and whether MEIv2 was positive or negative during the month when each passage quantile most often occurred, using the MEIv2 value for the two‐month period beginning in the passage month. We also identified years following El Niño or La Niña events as indicated by the National Weather Service (National Weather Service, 2023). We used two indices related to the NAM, the Arctic Oscillation (AO) and the North Atlantic Oscillation (NAO), retrieved from https://www.cpc.ncep.noaa.gov. For each metric of the NAM, we averaged across November–February (winter), March–May (spring), and August–October (fall). We also determined the AO and NAO values and whether the AO and NAO were positive or negative during the month when each passage quantile most often occurred.

### Models

#### Interannual variation in migratory passage phenologies

We used a Bayesian hierarchical framework to model the plasticity of bird migration timing to the interannual anomalies in environmental conditions, while accounting for long‐term trends in migration phenologies and environmental conditions and for climate oscillation modes (NAM and ENSO). We ran separate models for 10%, 50%, and 90% passage dates. Seasonal passage dates were modeled as a function of year, latitude, longitude, the interaction of latitude × longitude, the interannual anomalies in NAM and ENSO indices, and the interannual anomalies in environmental conditions. Models included random intercepts for station and random slopes for the effect of year by station. To select ENSO and NAM metrics, we tested each metric individually in a model that did not include interannual anomalies in environmental conditions, and we selected one ENSO and one NAM metric for each passage quantile that maximized the explained variation. For the models relating passage dates to interannual anomalies in environmental conditions, the interannual anomalies were themselves modeled as latent variables representing the difference between the observed environmental conditions and the expected conditions based on long‐term trends (for environmental conditions) or expected value (for climate oscillation mode indices, which were expected to fluctuate but not show linear trends over the period of study). We calculated standardized effect sizes by dividing the coefficient estimate for the effect of the interannual anomalies in each environmental condition by the SD of that condition in our data.

#### Long‐term trends in passage dates, environmental conditions during passage periods, and environmental conditions experienced by migrants on passage dates

We modeled long‐term trends in several response variables, including passage dates from 1995 to 2018, temperature and relative humidity on passage dates and during passage periods from 1995 to 2018, and EVI and LSP on passage dates and during passage periods from 2003 to 2018. We modeled each response variable as a function of latitude, longitude, the latitude × longitude interaction, and year, with a random intercept for station and a random slope for year by station.

If shifting migration phenology compensated for the impact of a changing environmental condition, we expected no long‐term trend in that condition on passage dates, even if a significant trend in that condition occurred within the passage periods, and that the 95% credible interval (CI) for the difference between these two trends would not overlap zero. To compare these trends, we subtracted the samples of the posterior distribution for the trends in the passage periods from the samples of the posterior distribution for trends on the passage dates.

#### Model fitting details

We completed all analyses with *rjags v 4‐15* (Plummer, 2023), using 5000 iterations in the adaptive phase and three chains, each with 15,000 burn‐in iterations and 18,000 samples from the posterior distribution, retaining every third sample. We checked model convergence using the *traceplot* function in the *coda v 0.19‐4* package (Plummer et al., 2006), confirming that all *ȓ* values were <1.05. All models were linear functions with normal error distributions. All prior distributions were normal distributions centered at 0 with a SD of 100, except the prior distributions for population means of random intercept terms, which were centered at the mean value of the response variable being modeled with a SD of 100 to aid with model convergence. We visually checked that all residuals were approximately normal, centered around zero, and had equal variance across all values of our variables of interest. For more details on modeling, see Appendix S1: Section S1: *Supplemental methods*. We found some heteroskedasticity in models of green‐up and dormancy, which we further explain and explore in Appendix S1: Section S2: *Supplemental analyses*, where we also report analyses for maturity and dormancy dates and for LSP analyses separated by land cover types.

## RESULTS

### Spring

**FIGURE 2:**
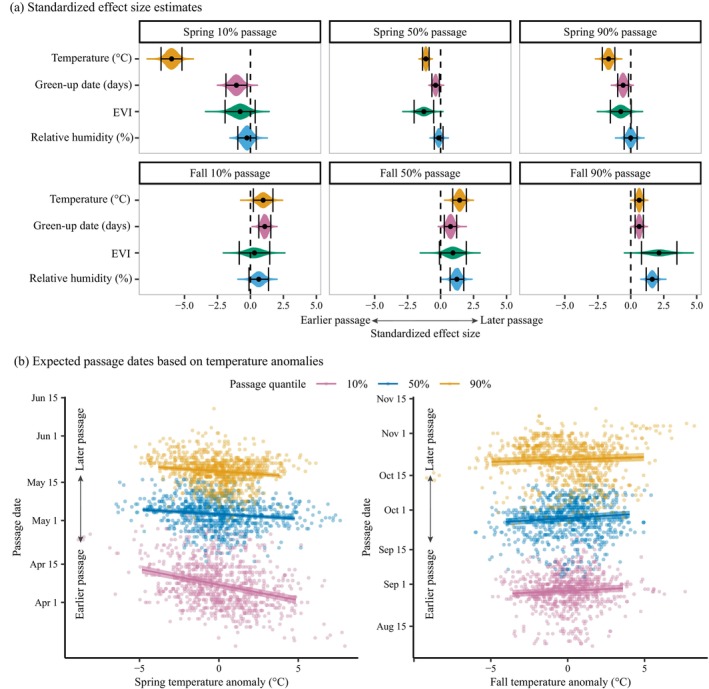
Effects of interannual anomalies in environmental conditions on annual passage dates. Interannual anomalies were defined as the observed values of the environmental conditions minus their expected values based on long‐term trends. Panel (a) shows standardized effect sizes for the interannual anomalies in each environmental condition/season. Shaded areas show the posterior probability distributions, and error bars show the 95% credible intervals (CIs). Panel (b) shows the passage dates versus interannual temperature anomalies. The lines and shaded areas show the expected values and 95% CIs for passage dates at 37° N and 96° W. EVI, enhanced vegetation index.

Interannual changes in spring migration phenology were most closely related to interannual anomalies in temperature for all passage quantiles, followed by green‐up date for 10% and 90% passage dates and EVI for 50% passage dates (Figure 2a, Table 1). Hereafter, values in parentheses indicate the 2.5% and 97.5% quantiles of the posterior probability distributions, and significance is reported when this 95% CI did not overlap zero. Passage dates occurred significantly earlier in years with higher temperature, with 10% passage advancing by 1.14 (0.99, 1.29) days/°C (Table 1). Only 50% passage occurred earlier in years with higher EVI, while none of the passage dates were significantly related to relative humidity (Figure 2a, Table 1). Unexpectedly, passage occurred significantly later in years with earlier green‐up, delaying by 0.02–0.04 days per day advancement in green‐up (Table 1). The effect sizes for green‐up date anomalies were substantially smaller than those for temperature (Table 1). Interannual anomalies in climate oscillation modes also affected spring passage dates, though effect sizes were smaller than those for temperature. Spring 50% passage occurred earlier in La Niña years, and 90% passage occurred earlier when MEIv2 was positive in May, while all passage quantiles advanced when the AO mode was more positive (Table 1). Models including the effects of geographic variables, random effects for station, long‐term trends in passage, and climate oscillation modes explained 67%, 80%, and 70% of the variances in spring 10%, 50%, and 90% passage dates, respectively. The interannual anomalies in environmental conditions explained 31%, 13%, and 8% of the variance that remained after modeling the effects of all other variables (Appendix S1: Table S7).

**TABLE 1:** Coefficient estimates and standardized effect sizes for the effects of interannual anomalies in environmental conditions on passage dates.

	Passage quantile					
	Spring 10%	Spring 50%	Spring 90%	Fall 10%	Fall 50%	Fall 90%
Standardized effect sizes^a^						
Temperature	**−5.98 (−6.78, −5.19)**	**−1.44 (−1.76, −1.12)**	**−1.51 (−1.94, −1.08)**	**0.97 (0.22, 1.71)**	**1.25 (0.8, 1.69)**	**0.64 (0.32, 0.96)**
Relative humidity	−0.26 (−0.96, 0.44)	−0.16 (−0.52, 0.20)	−0.01 (−0.48, 0.46)	0.63 (−0.11, 1.37)	**1.17 (0.68, 1.67)**	**1.63 (1.17, 2.08)**
EVI	−0.78 (−1.90, 0.36)	−1.08 (−1.70, −0.45)	−0.80 (−1.64, 0.03)	0.31 (−0.85, 1.46)	1.04 (−0.10, 2.16)	**2.14 (0.81, 3.50)**
Green‐up/dormancy	**−1.06 (−1.86, −0.26)**	**−0.49 (−0.85, −0.12)**	**−0.58 (−0.99, −0.17)**	**1.09 (0.64, 1.54)**	**0.58 (0.22, 0.95)**	**0.64 (0.33, 0.95)**
*ENSO index* ^b^	*−0.08 (−0.56, 0.4)*	***−0.60 (−0.94, −0.25)***	***−0.83 (−1.30, −0.35)***	*−0.57 (−1.27, 0.12)*	***−0.81 (−1.40, −0.23)***	***−0.98 (−1.51, −0.46)***
*NAM index* ^c^	***−0.70 (−1.18, −0.22)***	***−0.31 (−0.49, −0.13)***	***−0.26 (−0.46, −0.05)***	***2.76 (1.78, 3.75)***	*0.27 (−0.08, 0.62)*	***0.51 (0.21, 0.82)***
Absolute effect sizes^d^						
Temperature (days/°C)	**−1.14 (−1.29, −0.99)**	**−0.34 (−0.41, −0.26)**	**−0.39 (−0.51, −0.28)**	**0.29 (0.07, 0.51)**	**0.38 (0.24, 0.51)**	**0.19 (0.1, 0.29)**
Relative humidity (days/%)	−0.02 (−0.07, 0.03)	−0.01 (−0.04, 0.01)	0.00 (−0.04, 0.03)	0.05 (−0.01, 0.10)	**0.09 (0.05, 0.13)**	**0.12 (0.09, 0.16)**
EVI (days/1 unit EVI)	−9.55 (−23.64, 4.44)	−10.63 (−16.84, −4.42)	−7.47 (−15.21, 0.31)	2.58 (−7.09, 12.26)	8.68 (−0.82, 18.11)	**17.97 (6.77, 29.32)**
Green‐up/dormancy (days/day)	**−0.04 (−0.07, −0.01)**	**−0.02 (−0.03, −0.00)** ^e^	**−0.02 (−0.04, −0.01)**	**0.05 (0.03, 0.07)**	**0.03 (0.01, 0.05)**	**0.03 (0.02, 0.05)**
ENSO index**	*−0.08 (−0.56, 0.40)*	***−0.60 (−0.94, −0.25)***	***−0.83 (−1.30, −0.35)***	***−0.57 (−1.27, 0.12)***	***−0.81 (−1.40, −0.23)***	***−0.98 (−1.51, −0.46)***
NAO index***	***−0.81 (−1.36, −0.25)***	***−0.47 (−0.74, −0.2)***	***−0.34 (−0.63, −0.07)***	***2.76 (1.78, 3.75)***	*0.38 (−0.12, 0.87)*	***0.72 (0.29, 1.15)***

*Note*: Effect sizes are the expected change in passage date per SD change in the environmental condition (roman typeface) or climate oscillation modes (italics). Parentheses show the 95% credible intervals. Values are bolded if credible intervals do not overlap zero.

Abbreviation: EVI, enhanced vegetation index.

^a^ Change in passage/1 SD change in environmental variable.

^b^ ENSO (El Niño Southern Oscillation) indices selected: spring 10%: MEIv2 (Multivariate ENSO Index version 2) mean winter; spring 50%: La Niña year; spring 90%: MEIv2 positive in May; fall 10%: La Niña year; fall 50%: La Niña year; fall 90%: El Niño year. In cases where the presence/absence of a La Niña or El Niño year was selected, the standardized effect size is equal to the effect of those conditions being present.

^c^ NAM (Northern Annular Mode) indices selected: spring 10%: AO (Arctic Oscillation) value in April; spring 50%: AO mean spring; spring 90%: AO mean May; fall 10%: North Atlantic Oscillation (NAO) positive in August; fall 50%: NAO mean fall; fall 90%: NAO mean fall. In cases where AO is positive the standardized effect size is equal to the effect of AO being positive.

^d^ Change in passage date/1 unit change in environmental variable.

^e^ −0.00 indicates a very small negative number >−0.005.

**FIGURE 3:**
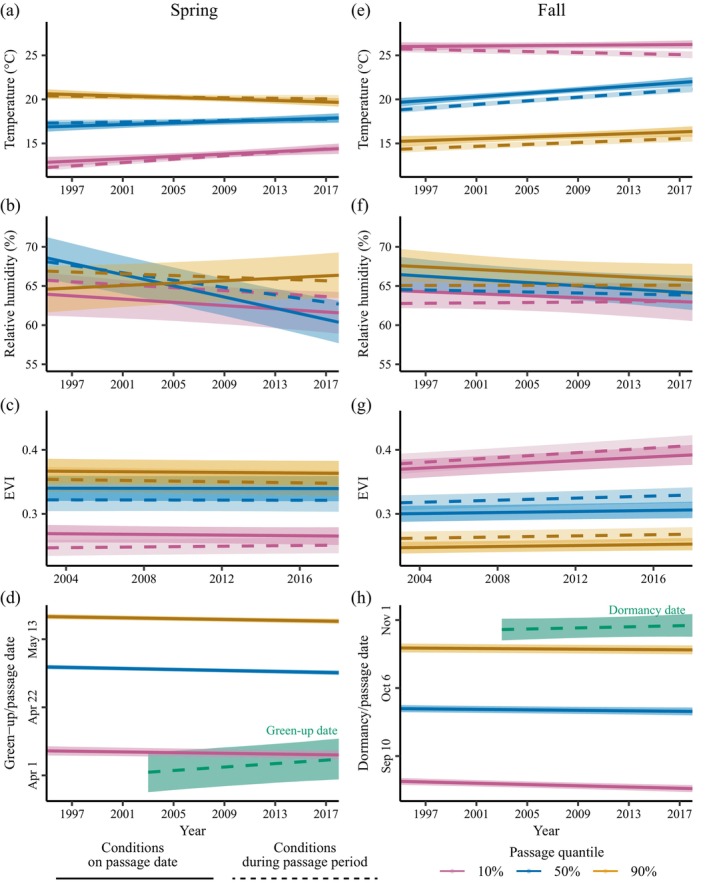
Decadal trends in environmental conditions on passage dates (solid lines) and during passage periods (dashed lines). Shaded areas represent 95% credible intervals (CIs) for conditions on passage dates (darker shading) and during passage periods (lighter shading). If migration phenologies shifted to compensate for changes in environmental conditions (Figure 1d/e), we expected conditions on passage dates (solid lines) to remain unchanged or have a shallower slope than overall trends in conditions during the passage periods (dashed lines). Panels (d/h) include trend lines for green‐up and dormancy dates. Predictions are shown for 37° N and 96° W. EVI, enhanced vegetation index.

Over the study period, spring migration advanced significantly for 50% and 90% passage by 0.77 (0.47, 1.07) and 0.63 (0.30, 0.95) days/decade, respectively (Table 2). Early (10%) passage also advanced, but the 95% CI overlapped 0. The advancement in passage date was not enough to prevent temperatures on 10% passage dates from increasing by 0.67°C/decade, following a trend similar to the warming that occurred during the passage period (Table 2, Figure 3a, Appendix S1: Figure S2). Temperatures on 50% passage dates also increased significantly by 0.44 (0.06, 0.81)°C/decade, while temperatures on 90% passage dates decreased significantly by 0.42 (0.07, 0.77)°C/decade, despite no significant change in temperature during the respective passage periods (Table 2). Relative humidity decreased on 10% and 50% passage dates by −1.03 (−2.25, 0.10) and −3.57 (−4.69, −2.45) %/decade, following trends that did not differ significantly from the trends during the respective passage periods. No significant changes in EVI occurred either during any of the spring passage periods or on the passage dates. Green‐up dates occurred 2.67 (0.46, 4.84) days/decade later, and proximity of passage date to green‐up (green‐up date minus passage date) increased for all quantiles by approximately 3 days, meaning that migrants experienced earlier land surface phenophases and passed sooner before or at a shorter interval after the green‐up phenophase transition (Figure 3d/h, Table 2).

**TABLE 2:** Decadal trends in passage dates (first row), environmental conditions on passage dates (italicized), environmental conditions during passage periods, and trends on passage dates minus trends during passage periods.

		Passage quantile					
		Spring 10%	Spring 50%	Spring 90%	Fall 10%	Fall 50%	Fall 90%
Passage date trends		−0.54 (−1.13, 0.03)	**−0.77 (−1.07, −0.47)**	**−0.63 (−0.97, −0.30)**	**−1.20 (−1.7, −0.70)**	−0.46 (−0.94, 0.03)	−0.32 (−0.69, 0.05)
Temp. (°C)	*On passage date*	***0.67 (0.25, 1.08)***	***0.44 (0.06, 0.81)***	***−0.42 (−0.77, −0.07)***	*0.10 (−0.17, 0.39)*	***1.02 (0.65, 1.36)***	***0.48 (0.08, 0.88)***
	In passage period	**0.96 (0.74, 1.17)**	0.20 (−0.02, 0.42)	−0.15 (−0.37, 0.06)	**−0.29 (−0.44, −0.13)**	**1.02 (0.83, 1.21)**	**0.55 (0.34, 0.76)**
	Diff. in trends	−0.29 (−0.78, 0.19)	0.24 (−0.21, 0.68)	−0.27 (−0.68, 0.15)	**0.39 (0.06, 0.72)**	0.00 (−0.41, 0.39)	−0.06 (−0.52, 0.38)
Relative humidity (%)	*On passage date*	*−1.03 (−2.25, 0.10)*	***−3.57 (−4.69, −2.45)***	*0.77 (−0.3, 1.83)*	*−0.62 (−1.78, 0.5)*	*−1.02 (−2.16, 0.08)*	*−0.8 (−2.09, 0.33)*
	In passage period	**−0.95 (−1.52, −0.4)**	**−2.35 (−3.04, −1.68)**	−0.56 (−1.13, 0.05)	0.12 (−0.51, 0.7)	−0.32 (−0.92, 0.3)	0.01 (−0.6, 0.65)
	Diff. in trends	−0.09 (−1.4, 1.19)	−1.22 (−2.54, 0.09)	**1.33 (0.12, 2.54)**	−0.74 (−2.04, 0.55)	−0.7 (−1.95, 0.53)	−0.82 (−2.26, 0.46)
Enhanced vegetation index	*On passage date*	*−0.003 (−0.01, 0.004)*	*0.000 (−0.006, 0.006)*	*−0.002 (−0.008, 0.004)*	***0.015 (0.008, 0.024)***	*0.004 (−0.001, 0.009)*	*0.004 (−0.001, 0.008)*
	In passage period	0.003 (−0.002, 0.008)	0.000 (−0.006, 0.005)	−0.004 (−0.010, 0.001)	**0.019 (0.012, 0.026**)	**0.008 (0.003, 0.013)**	**0.004 (0.001, 0.008)**
	Diff. in trends	−0.005 (−0.014, 0.003)	0.000 (−0.008, 0.008)	0.002 (−0.006, 0.010)	−0.004 (−0.014, 0.006)	−0.004 (−0.012, 0.003)	−0.001 (−0.007, 0.005)
Green‐up/dormancy (GU/D) dates (days)	*GU/D date—Passage date*	***3.14 (0.77, 5.51)***	***3.51 (1.11, 5.91)***	***3.35 (0.93, 5.59)***	*0.52 (−2.24, 3.24)*	*−0.62 (−3.32, 2.18)*	*−0.07 (−2.89, 2.71)*
	GU/D date	2.67 (0.46, 4.84)	2.67 (0.46, 4.84)	2.67 (0.46, 4.84)	1.05 (−1.45, 3.53)	1.05 (−1.45, 3.53)	1.05 (−1.45, 3.53)
	Diff. in trends	0.46 (−2.83, 3.73)	0.84 (−2.51, 4.06)	0.7 (−2.54, 3.9)	−0.56 (−4.26, 3.21)	−1.67 (−5.4, 2.14)	−1.07 (−4.79, 2.73)

*Note*: Values in parentheses are 95% credible intervals (CIs). Bold values indicate that the 95% CI does not overlap zero. For the rows labeled “Diff. in trends,” a CI that does not overlap zero indicates that trend in the environmental condition on the passage date was significantly different than the trend during the respective passage periods, while mean value near zero indicates that the trend in the environmental condition on the passage date was similar to the trend during the respective passage period.

### Fall

Fall migratory passage occurred later in years with higher temperatures, higher relative humidity, and later dormancy dates (Figure 2a, Table 1). The effect size for temperature was smaller in the fall than in the spring, with each fall migration quantile advancing significantly, but by <0.5 days/°C. Among fall passage quantiles, temperature had the largest effect on 50% passage dates. Passage also advanced significantly in years with later dormancy, and the effect size for dormancy was greater than that of temperature for 10% passage and equal to that of temperature for 90% passage (Figure 2a, Table 1). Fall 50% and 90% passage occurred later in years with higher relative humidity. Among all effect sizes estimated for environmental conditions in the fall, the effect of EVI on 90% passage was the largest, with passage delayed by 2.14 (0.81, 3.50) days per SD in EVI. When considering climate oscillation modes, the NAM index showed an even larger effect on 10% passage, with passage occurring 2.76 (1.77, 3.75) days later when the NAO was positive in the fall. Fall 50% passage advanced slightly in La Niña years, while 90% passage advanced slightly in El Niño years (Table 1). Models including the effects of geographic variables, random effects for station, long‐term trends in passage, and climate oscillation modes explained 65%, 77%, and 86% of the variances in spring 10%, 50%, and 90% passage dates, respectively. The interannual anomalies in environmental conditions explained 5%, 12%, and 14% of the variance that remained after modeling the effects of all other variables (Appendix S1: Table S7).

In the fall, only 10% passage showed a significant decadal trend, occurring 1.20 (0.70, 1.70) days/decade earlier (Table 2). Fall 50% and 90% passage also occurred earlier, but not significantly so. The trends in environmental conditions experienced by fall migrants mirrored the trends that occurred during the passage periods (Table 2, Figure 3, Appendix S1: Figure S2). There were no significant differences between the passage date and passage period trends, except that temperature during the 10% passage period became significantly cooler by 0.29 (0.13, 0.44)°C/decade, while temperatures on 10% passage dates showed no significant change (Figure 3e, Table 2). Temperatures increased by 1.02 (0.65, 1.36)°C/decade on 50% passage dates and by 0.48 (0.08, 0.88)°C/decade on 90% passage dates. EVI increased significantly during all three passage periods and on 10% passage dates. EVI trends for 50% and 90% passage dates were not significantly different from zero, but they were also not significantly different from the significant trends that occurred during the passage period (Table 2). There were no significant trends in relative humidity during passage periods or on passage dates and no significant trends in dormancy dates or passage proximity to dormancy dates.

## DISCUSSION

### Summary

Our research fills two important knowledge gaps regarding avian migration phenology by comparing the effects of multiple drivers on migration timing and including the fall season, which is understudied relative to spring (Gallinat et al., 2015). Moreover, we provide the first analysis of decadal trends in multiple environmental conditions experienced by the assemblage of migrants en route. We found that temperature was the primary driver of interannual changes to migration phenology in the spring, with much stronger effects than green‐up date, and there were multiple important drivers in the fall. Temperatures increased on three of the six passage phenology dates, and shifting migration phenologies did not keep pace with most changes in environmental conditions, following a “no compensation” scenario (Figure 1f). Our full models—including geographic variables, random effects, long‐term trends in passage dates, and interannual anomalies in environmental conditions—explained from 67% to 88% of the variance in each passage quantile date (Appendix S1: Table S7), demonstrating a comprehensive understanding of what drives variation in Central Flyway migration phenology across time and space.

### Spring

Spring migrants altered their annual migration phenologies in response to changes in environmental conditions, especially temperature. Studies of interannual variation in migratory passage or arrival dates have considered migrants' response to either green‐up or temperature, but not both (Ellwood et al., 2010; Horton et al., 2020; Horton, Morris, et al., 2023; Marra et al., 2005; Youngflesh et al., 2021). When the correlations between the interannual anomalies in migration phenologies and temperatures were observed in previous studies, they were interpreted as a proxy for a response to resource availability more broadly (Horton et al., 2020; Horton, Morris, et al., 2023). When we considered temperature in the same model with EVI and green‐up date as surrogates for resources, temperature was overwhelmingly the strongest predictor in the spring. Passage phenologies may respond more strongly to temperature than to the other environmental variables because temperature may correlate more directly with insect abundance, or it may be a more consistent cue about the conditions farther north because vegetation conditions can vary over relatively small distances (Brooks et al., 2020). Additionally, populations and species may have responded differently to changes in other environmental variables such that the phenologies observed via radar did not change even though some groups migrated earlier or later.

In our results, the response to green‐up date was in the opposite of the expected direction—passage occurred earlier in years with later green‐up—though the confidence intervals may be underestimated due to heteroskedasticity in the residuals with respect to green‐up date anomalies. This pattern disappeared or showed the expected relationship (earlier passage on years with earlier green‐up) for most quantiles when we modeled eastern (≤97° W) and western regions separately to address the heteroskedasticity (Appendix S1: Table S3) but remained significant for 10% passage in the east and 90% passage in the west. Decoupling of passage dates from green‐up in the Central Flyway may be due to the prevalence of cropland, where interannual changes to the vegetated land surface are heavily influenced by crop choice, agronomic practices, and irrigation patterns (Zhang et al., 2019). Green‐up dates in woody areas, which are likely to host a high density of migrants on stopover (Horton, Buler, et al., 2023), did not significantly affect passage dates (Appendix S1: Table S1).

The climate oscillation modes affected spring passage phenologies, although effect sizes were smaller than those for temperature. Migrants have been found to arrive on the Gulf Coast in better condition following La Niña years (Paxton et al., 2014), which could explain their advanced passage through the Central Flyway on years following a La Niña event (for 10% passage) and when MEIv2 was positive in May (for 90% passage). Among the NAM indices, AO indices were consistently selected over NAO indices, despite the latter being more commonly associated with bird migration phenology (Haest et al., 2018). A more comprehensive analysis of the climate teleconnections could reveal complex relationships with migration phenologies (Dezfuli et al., 2022) but extends beyond the scope of our study.

Spring migration advanced by more than 0.5 days/decade for all three migration quantiles (though the trend in 10% passage dates was not significant), consistent in direction but smaller in magnitude than the advances observed in other studies (Horton et al., 2020; Romano et al., 2023). Our estimate was consistent with the trend of 0.6 days/decade advancement estimated from bird‐building collision data in Chicago for median passage (Zimova et al., 2021). Chicago lies within the US Central Flyway, and advances in this region may be occurring more slowly than in other flyways.

Shifts in spring migration phenologies did not compensate for long‐term changes in the environmental conditions experienced on migratory passage dates. Temperatures increased on 10% passage dates and decreased on 90% passage dates, following the same trends observed during the respective passage periods. We observed decreased relative humidity on 10% and 50% passage dates, which could lead to higher water loss rates and reduced body condition for birds (Gerson & Guglielmo, 2011; Klaassen, 2004) and lower survival times for insects (Kleynhans & Terblanche, 2011). Trends in migration phenologies also intensified the change in LSP experienced by migrants: all migration quantiles occurred closer to the green‐up date by more than three days per decade due to the combination of advancing passage dates and delaying green‐up dates within the flyway, especially in areas west of 97° W longitude (Appendix S1: Table S6). Delays in the green‐up date could be explained by changes in cropping patterns in the Great Plains (Zhang et al., 2019) or by declining precipitation in grassland systems (Post et al., 2022). Robertson et al. (2024) found that across North America, green‐up dates are advancing, and that bird migration phenology has not kept pace—birds are passing later relative to green‐up. Although the green‐up date trends within the Central Flyway appear to diverge from other parts of North America, our results point to a similar conclusion: changes to migration phenology have not prevented changes to the timing of migratory passage relative to green‐up.

### Fall

Unlike in spring—when temperature remained the primary predictor of passage dates even when other environmental conditions were considered—other conditions were nearly as or more important than temperature for explaining fall passage phenologies. Previous studies of fall migration in North America have found that some species depart or pass through stopover areas later on warmer years, but these studies were limited to short‐distance migrants (Brisson‐Curadeau et al., 2019) or warblers (Horton, Morris, et al., 2023) and did not consider the many other environmental conditions that may affect fall migration phenology. All fall migration quantiles occurred earlier in warmer years with later dormancy. Fall 50% and 90% passage occurred later on years with higher relative humidity, and 90% passage occurred later on years with higher EVI. These years may have had higher fall resource availability, which may allow birds to remain on breeding sites or stopover grounds longer. Their response to vegetation phenological status and biomass in the fall, but not in the spring, may have occurred because many fall migrants incorporate fruit in their diet (Mudrzynski & Norment, 2013; Parrish, 2000), which allows them to gain fat more quickly (Parrish, 1997). A direct index of fruiting phenology, not currently available at the flyway scale, may better explain annual migration phenologies than any of the environmental variables we evaluated.

The interannual anomalies model estimated a 2.76 (1.78, 3.75) day delay in 10% passage when NAO was positive in August, which was the largest effect size of any climate oscillation index. This effect could explain why fall 10% passage occurred earlier over time: NAO was positive in August six times between 1995 and 2006 and only twice between 2007 and 2018. Fall 90% passage was also delayed when mean August–October NAO values were higher, highlighting a potential role for the NAM to shape fall migration phenology.

Our finding that fall 10% passage has advanced in recent decades is supported by other evidence, whereas the non‐significant advancing trends in fall 50% and 90% passage dates were inconsistent with previous studies. Evidence from building collisions in Chicago also showed that early‐migrating species advanced their passage (Zimova et al., 2021), which the authors attributed to earlier breeding and molt completion in species that do not produce a second brood (Mitchell et al., 2012; Saino et al., 2017; van Wijk et al., 2017). The advance of early fall migration may additionally or alternatively be attributed to temperature trends during the fall 10% passage period, which declined by 0.29 (0.13, 0.44)°C/decade. While median and late migrants in Chicago showed delays between 1978 and 2016, our radar estimates for 50% and 90% passage did not change between 1995 and 2018 perhaps due to differences in study period length. A previous analysis of the same radar data found that fall migration advanced significantly for all quantiles and estimated 0.34 ± 0.18 days/decade advancement for 50% passage dates. Our estimate here of 0.46 (−0.94, 0.03) days/decade advancement is similar in magnitude but non‐significant likely because we used a more conservative statistical approach that accounted for the repeated sampling of the same stations across years using a random effect.

The interannual adjustments (for all migration quantiles) and long‐term advancement (at 10% passage dates) did not compensate for the changes in environmental conditions during the fall migration season; trends in the conditions experienced on fall passage dates closely matched the mean trends within their passage periods. The one exception was that temperature on early fall passage dates remained consistent across the study period despite declining temperatures during the 10% passage period. Migrating earlier may have allowed early migrants to reduce exposure to cooler temperatures. Median and late fall migration dates, on the other hand, did not adjust to increasing temperatures within those passage periods; these passage dates experienced temperature increases of 1.02 (0.65, 1.36)°C and 0.48 (0.08, 0.88)°C per decade, respectively. This failure to adjust to moderately increasing fall temperatures suggests that migrants will continue to experience increasing temperatures as changing climates warm. No other changes to environmental conditions on passage dates in the fall were significant except for the increase in EVI for 10% passage. Neither dormancy date nor passage proximity to dormancy date changed during the study period, though changes in fruiting phenologies may have occurred if dormancy and fruiting phenology were not correlated. Some species may delay passage enough to reduce the increase in temperature that they experience, but we did not find evidence for compensation in the assemblage of migratory birds detected by radar. Further research could couple WSR data with Motus or other wildlife tracking systems to understand species‐level variation in the trends in conditions experienced en route.

## CONCLUSION

The flyway‐scale perspective obtained from WSR data showed that assemblages of avian migrants shifted their annual migration phenologies in accordance with annual changes to some environmental conditions, especially spring temperatures, and advanced over time. However, these shifts were not enough to compensate for trends in environmental conditions. Avian migrants are exposed to rapidly changing environmental conditions over the course of two to three weeks as they migrate north or south through the US Central Flyway (Adams et al., 2024; Nemes et al. 2023), so decadal trends of up to 2°C warming, 5% decrease in relative humidity, or 3‐day delays in green‐up are unlikely to cause substantial physiological stress. However, continued environmental changes may expose birds to conditions not previously faced on migration, especially in locations where they already experience extreme conditions.

Furthermore, the northward/southward progression of avian migration may align temporally with regional resource peaks not captured by temperature or remotely sensed vegetation indices, and these conditions may also be changing over time more rapidly than bird migration phenologies. As spring migrants pass closer to green‐up in the Central Flyway, they may experience earlier phenologies of herbivorous insects (Bartomeus et al., 2011; Pöyry et al., 2018). Increasing temperatures on 10% passage may also have important consequences for aquatic insect availability for early migrants (Dallas & Ross‐Gillespie, 2015; Nash et al., 2023; Twining et al., 2018). Passage trends indicate that migrants may be arriving at breeding sites in the US Central Flyway closer to green‐up, which has been shown to correspond to decreased breeding productivity at the population level (Youngflesh et al., 2023). Considering the rising temperature trends during the 50% and 90% passage periods, fall fruiting may have advanced in the US Central Flyway (Menzel et al., 2006), but very little is known about how wild fruits respond to increasing temperatures. In New England, fruiting was found to occur later over time (~1 day later per decade between 1892 and 2012) and earlier in years with warmer springs for some species (Gallinat et al., 2018). These trends may be different in the grassland regions of the Central Flyway because vegetation phenology may be more affected by annual precipitation than temperature (Post et al., 2022). Changes to fruiting phenologies could have important implications for fall migrants because of their mutualistic relationship with fall‐fruiting plants (Gallinat et al., 2020; Stiles, 1980; Thompson & Willson, 1979). Further understanding of how climate change affects phenological synchrony between avian migrants' passage phenologies and the emergence or peak abundance of their prey items may become possible as technologies emerge to document macroecological phenomena for plants and insects (Crimmins et al., 2017; Van Klink et al., 2022).

Observed migration phenologies for the assemblage of migrants observed by WSR in the Central Flyway have not as yet adjusted to the environmental changes that have already occurred and will likely continue to change. Thus, future migratory cohorts may face conditions en route that reach critical points leading to higher mortality rates, lower rates of cohort recruitment, and diminishing populations of migratory birds.

## CONFLICT OF INTEREST STATEMENT

The authors declare no conflicts of interest.

## Supporting information


Appendix S1.

## Data Availability

The data and code that was generated and/or analyzed for this study are available on Figshare at https://doi.org/10.6084/m9.figshare.26050390 (Adams et al., 2025). Radar processing code and algorithms (Sheldon et al., 2019) are available on Zenodo at https://doi.org/10.5281/zenodo.3351749. Moderate Resolution Imaging Spectroradiometer (MODIS) vegetation product (MOD13A1 V6.1) was downloaded from NASA's Land Processes Distributed Active Archive Center (LP DAAC) at https://e4ftl01.cr.usgs.gov/ using the pymod python package (http://pymodis.org/). We selected 14 tiles: h08v04, h08v05, h08v06, h09v04, h09v05, h09v06, h10v04, h10v05, h10v06, h11v04, h11v05, h12v04, h12v05, and h13v04 from January 1, 2003, to January 1, 2018. The MODIS land cover dynamics product (MCD12Q2 V6.1) was ordered and downloaded via the Application for Extracting and Exploring Analysis Ready Samples (AppEEARS) tool at https://appeears.earthdatacloud.nasa.gov/; using Extract Area Sample, for start date January 1, 2003, and end date January 1, 2018, we selected two layers from the MCD12Q2.061 product “Greenup and Dormancy”; as a spatial extent, we used coordinates as follows: for upper left corner 50.414063° N and 110.179687° W, for bottom right corner 23.484375° N and 86.0625° W; as output details, we chose GeoTiff file format at native projection. North American Regional Reanalysis (NARR) data are available from the U.S. National Oceanic and Atmospheric Administration Physical Sciences Laboratory at https://downloads.psl.noaa.gov/Datasets/NARR/Dailies/monolevel/; for relative humidity, we downloaded rhum.2m.1995.nc through rhum.2m.2018.nc by clicking on each individual file, and for air surface temperature, we downloaded air.sfc.1995.nc through air.sfc.2018.nc.
