# Supplementary material for: Shifts in avian migration phenologies do not compensate for changes to conditions en route in spring and fall
Source: Ecology. 2025 May 18;106(5):e70110. doi: 10.1002/ecy.70110 (PMC12086353; doi:10.1002/ecy.70110)
Supplement: Supplementary file 1 — Appendix S1. [file ECY-106-e70110-s001.pdf]

Appendix S1

Shifts in avian migration phenologies do not compensate for changes to conditions *en route* in spring and fall

Journal: Ecology

Authors:

Carrie Ann Adams,

Monika A. Tomaszewska,

Geoffrey M. Henebry,

Kyle G. Horton

Section S1. Supplemental methods

Models

Inter-annual variation in migratory passage phenology

Comparing a time series of migration phenology to a time series of temperature or another variable, without detrending the data, may result in a significant correlation because both are changing over time in response to other drivers (McLean et al. 2022). We used a Bayesian hierarchical framework to model the effects of inter-annual changes in environmental conditions on bird migration phenology, while accounting for the long-term trends in environmental conditions, the long-term trends in migration phenology that were unrelated to these inter-annual changes, and climate oscillations (Northern Annular Mode (NAM) and El Niño Southern Oscillation (ENSO)).

We ran separate models for 10%, 50%, and 90% passage dates. We subtracted 2003 from the year variable so that year had values 0 (in 2003) to 15 (in 2018) and standardized latitude and longitude to mean zero and standard deviation one. We only included data from 2003 – 2018, the period during which both passage data and MODIS data from both the Terra and Aqua satellites were available.

$$(\textbf{Equation S1})\ \textbf{Long} - \textbf{term trends in environmental conditions}:$$

$$\boldsymbol{L_{j,s,l}} \sim \text{Normal}\big(\text{E}(L_{j,s,l}), \sigma^2_{L,l}\big)$$

$$\text{E}(L_{j,s,l}) = \alpha_{s,l} + \beta_{s,l} \times \boldsymbol{year_j} + \gamma_l \times \boldsymbol{lat_s} + \theta_l \times \boldsymbol{lon_s} + \delta_l \times \boldsymbol{lat_s} \times \boldsymbol{lon_s}$$

$$\alpha_{s,l} \sim N(\mu_{\alpha,l}, \sigma^2_{\alpha,l})$$

$$\beta_{s,l} \sim N(\mu_{\beta,l}, \sigma^2_{\beta,l})$$

At the first modeling level, a matrix of environmental conditions (**L**) was modeled as a function of year, latitude, longitude, and the latitude × longitude interaction. In this and all other models, the effects of latitude, longitude, and latitude × longitude are represented by γ, θ, and δ, respectively. **L** is a 3-dimensional matrix, including dimensions for year (*j*), station (s), and environmental condition (*l*). Each environmental condition was modeled with $\mathbf{L}_l$ a normal error distribution with standard deviation $\sigma_{L,l}$. For each environmental condition modeled, we included random intercept $\alpha_{s,l}$ for station and slopes $\beta_{s,l}$ for station/year to account for variation among sites in the environmental conditions in 2003 (the intercept) and the change over time (the slope). These random intercepts and slopes were drawn from normal distributions with means $\mu_{\alpha,l}$ and $\mu_{\beta,l}$, and standard deviations $\sigma_{\alpha,l}$ and $\sigma_{\beta,l}$.

$$(\textbf{Equation S2})\ \textbf{Inter} - \textbf{annual anomalies in environmental condtions}:$$

$$\boldsymbol{A_{j,s,l}} = \boldsymbol{L_{j,s,l}} - \text{E}(L_{j,s,l})$$

We modeled the inter-annual anomalies in environmental conditions (***A***) as a latent variable representing the difference between the observed and expected values of ***L***.

$$(\textbf{Equation S3})\ \textbf{Mean values for climate oscillations}:$$

$$\boldsymbol{C_{j,c}} \sim \text{Normal}(\text{E}(C_c), \sigma_C^2)$$

$$E(C_c) = \alpha_c$$

Because we did not expect climate oscillation indices, which vary over time, to show long-term trends, we modeled their mean values as a single expected value for each climate index across all years, with a normally distributed error structure. ***C*** represents 2-dimensional matrix of climate oscillations indices (*c*) each year (*j*).

$$(\textbf{Equation S4})\ \textbf{Inter} - \textbf{annual anomalies in climate oscillations}:$$

$$\boldsymbol{O_{j,c}} = \boldsymbol{C_{j,c}} - \text{E}(C_c)$$

We estimated the inter-annual anomaly for each climate oscillation index in each year ($\boldsymbol{O_{j,c}}$) as the observed minus the expected value.

$$(\textbf{Equation S5})\ \textbf{Passage quantile dates}:$$

$$\boldsymbol{P_{j,s}} \sim \text{Normal}(\text{E}(P_{j,s}), \sigma_P^2)$$

$$\text{E}(P_{j,s}) = \psi_s + \pi_s \times \boldsymbol{year_j} + \gamma \times \boldsymbol{lat_s} + \theta \times \boldsymbol{lon_s} + \delta \times \boldsymbol{lat_s} \times \boldsymbol{lon_s}$$

$$+ \sum_{k=1}^{\text{N}_\text{l}} \omega_l \times \boldsymbol{A_{s,j,l}} + \sum_{k=1}^{\text{N}_\text{c}} \eta_c \times \boldsymbol{O_{j,c}}$$

$$\psi_{s,p} \sim \text{Normal}(\mu_{\psi,P}, \sigma_{\varphi,P}^2)$$

$$\pi_{s,p} \sim \text{Normal}(\mu_{\pi,P}, \sigma_{\pi,P}^2)$$

We modeled passage quantile dates (***P***) as a function of year (to account for the long-term trends in passage phenologies), the inter-annual anomalies in the environmental conditions (***A***), and the inter-annual anomalies in climate oscillation indices (***O***). ***P*** is a 2-dimensional matrix, including dimensions for year (*j*) and station (*s*). Each passage quantile was modeled separately. The model included a normally-distributed random intercept $\psi_{s,p}$ and a random slope $\pi_{s,p}$ for each station/year to account for variation among radar stations in the passage dates in 2003 (the intercept) and the change over time (the slope). N_l is the number of environmental conditions and N_c is the number of climate oscillations included in the model. We calculated the additional variance in passage dates explained by the inter-annual anomalies in environmental conditions by subtracting the R^2 value for a model that did not include the effect of inter-annual anomalies in environmental conditions from the R^2 value of the full model. For more details on how R^2 was calculated, see Table S7. Standardized effect sizes for all inter-annual anomalies were calculated by dividing the estimated coefficient by the standard deviation in that inter-annual anomaly.

Long-term trends in passage dates, environmental conditions during passage periods, and environmental conditions experienced by migrants on passage dates

We modeled long-term trends in passage quantile dates from 1995 – 2018. We modeled the long-term trends environmental conditions on passage dates and during passage periods using data from 1995 – 2018 for temperature and relative humidity and data from 2003-2018 for EVI and land surface phenology. The first year of the modelling period was set to 0, and latitude and longitude were standardized to mean 0 and standard deviation 1.

$$(\textbf{Equation S6})\ \textbf{Long}-\textbf{term trends in passage dates}, \\ \textbf{environmental conditions experienced by migrants}, \\ \textbf{environmental conditions during passage periods}, \\ \textbf{and phenophase transition dates}:$$

$$\mathbf{r} \sim \mathrm{Normal}(\mathrm{E(r)}), \sigma_r^2)$$

$$E(\mathrm{r}) = \psi_s + \pi_s \times \boldsymbol{year}_j + \gamma \times \boldsymbol{lat}_s + \theta \times \boldsymbol{lon}_s + \delta \times \boldsymbol{lat}_s \times \boldsymbol{lon}_s$$

$$\psi_s \sim N(\mu_\psi, \sigma_\psi^2)$$

$$\pi_s \sim N(\mu_\pi, \sigma_\pi^2)$$

We modeled each response variable ***r*** as a function of latitude, longitude, their interaction, and year, with a random intercept (ψ) and slope (π) for each station. The intercept represented the expected value in year 0 and the intercept represented the change in the expected value per year. Each response variable was analyzed in a separate model. The response variables tested were passage quantile dates, environmental conditions on passage quantile dates, the means of the environmental conditions during passage periods, and phenophase transitions dates (green-up and dormancy dates).

Details of Bayesian modeling

We ran these analyses with *rjags v 4-15* (Plummer 2023), using 5,000 iterations in the adaptive phase and 3 chains, each with 15,000 burn-in iterations and 18,000 samples from the posterior distribution, retaining every third sample. Unless otherwise specified, all prior distributions were normal distributions centered at 0 with standard deviation 100. In equation S5, the prior distribution for the random intercept hyperparameter $\mu_{\psi,P}$ was a normal distribution centered at the mean passage quantile date across all years and stations and with standard deviation of 100. Similarly, in equation S6, the prior distribution for $\mu_{\psi,r}$ was a normal distribution centered at the mean value of the response variable and with standard deviation of 100. These informative priors for the population-level mean response variable aided with model convergence. We checked model convergence using the *traceplot* function in the *coda* v 0.19-4 package (Plummer et al. 2006), confirming that all $\hat{r}$ values were < 1.05. We visually checked that

all residuals were approximately normal, centered around zero, and had equal variance across all values of our variables of interest. We found some heteroskedasticity in models of green-up and dormancy, which we further explain and explore in Appendix S1 Section S2, *Supplemental analyses*.

Box S1. Model Variable Definitions

Matrix indices:

- j = year, 24 values for analyses including 1995-2018 (years 0-23) and 16 possible values for analyses including years 2003-2018 (years 0-15)
- s = station, 53 values representing radar station IDs
- l = environmental condition, 4 values (1 = temperature, 2 = relative humidity, 3 = EVI, 4 = green-up date for spring or dormancy date for fall)
- c = climate oscillation index, 2 values (1 = ENSO metric, 2 = NAM metric)

Response and predictor matrices/vectors:

- $\boldsymbol{L}$ = 3-dimensional matrix of environmental variables, with dimensions for year (j), station(s), and environmental condition (l)
- $\boldsymbol{C}$ = 2-dimensional matrix of climate oscillations indices, with dimensions for year (j) and climate oscillation index (c).
- $\boldsymbol{P}$ = 2-dimensional matrix of passage quantile dates, with dimensions for year (j), station(s)
- ***year*** = vector of observation years
- ***lat*** = vector of radar station latitudes
- ***lon*** = vector of radar station longitudes
- $\boldsymbol{r}$ = vector of a response variable for the long-term trend models (equation S6). Each response variable (environmental condition or passage quantile date) was analyzed in a separate model.

Latent variables:

- $\boldsymbol{A}$ = 3-dimensonal matrix of estimated anomalies in environmental variables, with dimensions for year (j), station(s), and environmental condition (l). $\boldsymbol{A}$ was calculated as the observed minus the expected environmental conditions.
- $\boldsymbol{C}$ = 2-dimensional matrix of estimated anomalies in climate oscillation indices, with dimeson for year (j) and climate oscillation index (c)

References

McLean N, Kruuk LEB, Van Der Jeugd HP, et al (2022) Warming temperatures drive at least half of the magnitude of long-term trait changes in European birds. Proc Natl Acad Sci USA 119:e2105416119. https://doi.org/10.1073/pnas.2105416119

Plummer M (2023) rjags: Bayesian Graphical Models using MCMC

Section S2. Supplemental analyses

Methods

We repeated all analyses involving green-up and dormancy dates, and passage proximity to these dates, separately for three aggregated land cover classes from the MCD12Q1 V6.1 Land Cover Dynamics product (500-m resolution), using an aggregated version of IGBP classification system: cropland (classes 12 and 14), woody (classes 1-8), and grassy (classes 9 and 10).

We also repeated the analyses for different phenophase transition dates, maturity (date when EVI2 first crossed 90% of the EVI2 amplitude) in the spring and senescence (date when EVI2 last crossed 90% of the EVI2 amplitude) in the fall.

We repeated the analyses in regions east and west of 97°W without the year 2012 (Figure S1). We did this step because the residuals were more variable west of 97°W (though centered around zero across the range of longitudes) in the models of interannual anomalies, long-term trends, and passage proximity to land surface phenology transition dates. We also found that 2012 was a year with particularly early green-up (Ault et al. 2013).

Results

Spring

The relationship between passage phenologies and inter-annual anomalies in green-up dates remained largely consistent across cropland and grassy landcover types, but there was no relationship in woody landcover types (Table S1). When maturity date anomalies were modeled in place of green-up date anomalies, passage occurred earlier on years with later maturity (Table S2). When stations west and east of 97°W were analyzed separately and 2012 was omitted, spring 10% passage in the east occurred 0.18 (0.04, 0.33) days earlier per day advancement in green-up (following the expected pattern), 50% passage was not significantly related to green-up date, and 90% passage occurred 0.06 (0.02, 0.11) days later (Table S3). In the western region, 10% passage was delayed by 0.05 (0.02, 0.08) days per day advancement in green-up, and 50% and 90% passage dates were not significantly related to anomalies in green-up date.

The trend in delayed green-up was present in all land cover types considered, though not significant for cropland or woody land cover types (Table S4). Proximity of passage date to green-up increased significantly in all land cover types (Table S4), meaning that migrants experienced earlier land surface phenophases and passed sooner before or at a shorter interval after the green-up phenophase transition. Maturity dates also occurred later over time, and passage occurred sooner before maturity date (Table S5). When stations west and east of 97°W were analyzed separately and 2012 was omitted, the

trend of increasing proximity to green-up date was stronger in the western region. The trends were weaker in the eastern region and it was not significant in the eastern region for 10% passage (Table S6). Proximity of 50% passage date to green-up date increased by 6.79 (2.57, 11.25) days/decade in the western region and 2.1 (0.58, 3.6) days/decade in the eastern region.

Fall

Fall migration was delayed on years with later dormancy, and this pattern persisted for cropland and grassy landcover types, but not woody (Table S1). Passage also occurred later on years with later senescence, though the delay in 10% passage was not significant (Table S2). There were no significant effects of dormancy date on passage dates when eastern (<= 97°W) and western regions were modeled separately and the year 2012 was excluded (Table S3).

There were no significant dormancy dates trends in any land cover types (Table S4). Although senescence date occurred 3.39 (0.79, 5.98) days/decade later, passage proximity to senescence date did not change, and the difference between these two trends was not significant (Table S5). There were no significant trends in dormancy date or passage proximity to dormancy date when analyzing the eastern and western parts of the flyway separately and excluding the year 2012 (Table S6).

Figure S1. Locations of weather surveillance radar stations (dots) and 100-km radar zones (circles) within the Central Flyway. Solid lines represent the longitudinal boundaries defining the Central Flyway (87°W to 106°W). Dashed lines represent 97°W, where we divided the data east/west of this line for additional analyses of green-up/dormancy dates.

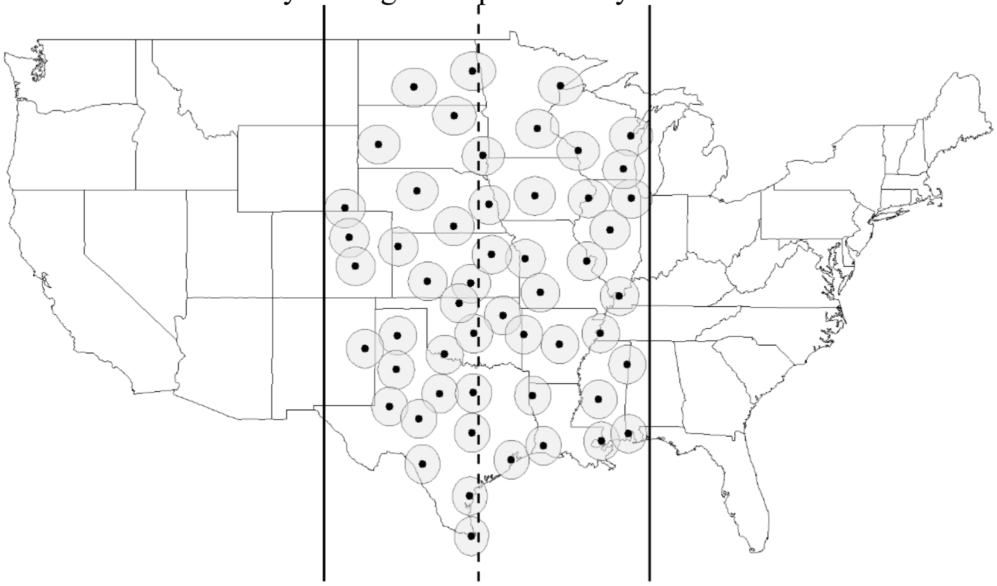

Table S1. Standardized effect sizes for effect of interannual anomalies in green-up and dormancy date in different classifications of land cover on passage dates. Parentheses show the 95% credible intervals. Values are bolded if credible intervals do not overlap zero.

	Spring 10%	Spring 50%	Spring 90%	Fall 10%	Fall 50%	Fall 90%
All pixels	**-1.06 (-1.86, -0.26)**	**-0.49 (-0.85, -0.12)**	**-0.58 (-0.99, -0.17)**	**1.09 (0.64, 1.54)**	**0.58 (0.22, 0.95)**	**0.64 (0.33, 0.95)**
Cropland	-0.82 (-1.67, 0.02)	**-0.65 (-1.06, -0.26)**	**-1.01 (-1.48, -0.54)**	**1.34 (0.71, 1.95)**	**0.76 (0.27, 1.23)**	**0.78 (0.37, 1.19)**
Grassy	**-0.66 (-1.32, -0.02)**	**-0.36 (-0.66, -0.05)**	**-0.38 (-0.72, -0.03)**	**0.86 (0.45, 1.26)**	**0.54 (0.21, 0.86)**	**0.56 (0.29, 0.84)**
Woody	0.28 (-0.33, 0.88)	-0.04 (-0.35, 0.28)	-0.18 (-0.58, 0.23)	0.22 (-0.25, 0.68)	0.20 (-0.17, 0.58)	-0.01 (-0.35, 0.33)

Table S2. Standardized effect sizes for effect of green-up and maturity (in the spring) and dormancy and senescence (in the fall) when included in separate versions of the interannual anomaly models. Parentheses show the 95% credible intervals. Values are bolded if credible intervals do not overlap zero.

Passage quantile	Green-up date (spring) or dormancy date (fall)		Maturity date (spring) or senescence date (fall)	
	days/ day	days/ sd	days/ day	days/ sd
Spring 10%	**-0.04 (-0.07, -0.01)**	**-1.06 (-1.86, -0.26)**	**-0.06 (-0.08, -0.03)**	**-1.47 (-2.14, -0.82)**
Spring 50%	**-0.02 (-0.03, -0.00**)**	**-0.49 (-0.85, -0.12)**	**-0.02 (-0.03, -0.00*)**	**-0.42 (-0.75, -0.10)**
Spring 90%	**-0.02 (-0.04, -0.01)**	**-0.58 (-0.99, -0.17)**	**-0.02 (-0.03, -0.00*)**	**-0.46 (-0.84, -0.09)**
Fall 10%	**0.05 (0.03, 0.07)**	**1.09 (0.64, 1.54)**	0.05 (-0.01, 0.10)	0.63 (-0.11, 1.37)
Fall 50%	**0.03 (0.01, 0.05)**	**0.58 (0.22, 0.95)**	**0.09 (0.05, 0.13)**	**1.17 (0.68, 1.67)**
Fall 90%	**0.03 (0.02, 0.05)**	**0.64 (0.33, 0.95)**	**0.12 (0.09, 0.16)**	**1.63 (1.17, 2.08)**

**-0.00 indicates a very small negative number > -0.005

Table S3. Coefficient estimates and standardized effect sizes for the effects of interannual anomalies in environmental conditions on passage dates when modeled separately west and east of 97°W, with the year 2012 removed. Parentheses show the 95% credible intervals. Values are bolded if credible intervals do not overlap zero.

(a) West

Passage quantile	Temperature		Relative humidity		EVI		Green-up date (spring) or dormancy date (fall)	
	days/ °C	days/ sd	days/%	days/sd	days/EVI	days/sd	days/day	days/sd
Spring 10%	**-0.83 (-1.02, -0.65)**	**-4.33 (-5.29, -3.37)**	0.00 (-0.05, 0.06)	0.04 (-0.72, 0.79)	-13.02 (-31.12, 4.73)	-0.91 (-2.19, 0.33)	**-0.05 (-0.08, -0.02)**	**-1.44 (-2.38, -0.50)**
Spring 50%	**-0.43 (-0.57, -0.29)**	**-1.95 (-2.59, -1.32)**	-0.03 (-0.07, 0.00*)	-0.49 (-1.0, 0.03)	-1.55 (-12.22, 9.19)	-0.13 (-0.99, 0.74)	-0.01 (-0.03, 0.01)	-0.19 (-0.77, 0.42)
Spring 90%	**-0.4 (-0.65, -0.15)**	**-1.68 (-2.74, -0.61)**	0.04 (-0.02, 0.11)	0.60 (-0.31, 1.52)	-2.71 (-15.82, 10.28)	-0.21 (-1.25, 0.81)	0.00 (-0.02, 0.03)	0.12 -0.63, 0.88)
Fall 10%	0.33 (-0.09, 0.76)	1.09 (-0.29, 2.48)	0.04 (-0.06, 0.15)	0.47 (-0.62, 1.59)	-15.42 (-32, 0.99)	-1.32 (-2.74, 0.09)	0.03 (-0.00, 0.06)	0.60 (-0.05, 1.24)
Fall 50%	**0.30 (0.08, 0.53)**	**0.99 (0.25, 1.74)**	**0.11 (0.05, 0.16)**	**1.14 (0.54, 1.74)**	-3.26 (-18.7, 12.38)	-0.28 (-1.6, 1.06)	0.02 (-0.01, 0.04)	0.34 (-0.18, 0.86)
Fall 90%	**0.18 (0.03, 0.34)**	**0.59 (0.08, 1.1)**	**0.14 (0.09, 0.18)**	**1.45 (0.95, 1.94)**	**17.43 (1.19, 33.63)**	**1.49 (0.1, 2.88)**	0.01 (-0.01, 0.03)	0.14 (-0.3, 0.57)

(b) East

Passage quantile	Temperature		Relative humidity		EVI		Green-up date (spring) or dormancy date (fall)	
	days/ °C	days/ sd	days/%	days/sd	days/EVI	days/sd	days/day	days/sd
Spring 10%	**-1.20 (-1.49, -0.90)**	**-5.80 (-7.22, -4.33)**	-0.09 (-0.2, 0.03)	-0.57 (-1.29, 0.16)	26.66 (-3.60, 56.75)	2.17 (-0.29, 4.62)	**0.18 (0.04, 0.33)**	**3.77 (0.92, 6.66)**
Spring 50%	**-0.34 (-0.45, -0.23)**	**-1.29 (-1.70, -0.88)**	0.01 (-0.04, 0.05)	0.04 (-0.25, 0.33)	-15.62 (-24.55, -6.67)	-1.49 (-2.35, -0.64)	-0.03 (-0.08, 0.01)	-0.71 (-1.7, 0.29)
Spring 90%	**-0.37 (-0.46, -0.27)**	**-1.24 (-1.56, -0.93)**	-0.03 (-0.06, 0.01)	-0.16 (-0.4, 0.08)	-20.55 (-29.9, -11.07)	-1.92 (-2.8, -1.04)	**-0.06 (-0.11, -0.02)**	**-1.31 (-2.17, -0.46)**
Fall 10%	0.19 (-0.06, 0.45)	0.62 (-0.18, 1.45)	0.06 (-0.01, 0.13)	0.48 (-0.08, 1.03)	**21.56 (6.69, 36.65)**	**1.36 (0.42, 2.31)**	-0.04 (-0.12, 0.04)	-0.37 (-1.20, 0.46)
Fall 50%	**0.48 (0.29, 0.67)**	**1.55 (0.94, 2.15)**	**0.10 (0.03, 0.16)**	**0.76 (0.25, 1.26)**	**24.80 (9.81, 39.6)**	**1.56 (0.62, 2.49)**	-0.03 (-0.11, 0.05)	-0.29 (-1.15, 0.57)
Fall 90%	**0.25 (0.12, 0.39)**	**0.81 (0.38, 1.24)**	**0.11 (0.05, 0.16)**	**0.84 (0.41, 1.26)**	**35.43 (15.04, 55.63)**	**2.23 (0.95, 3.5)**	-0.02 (-0.09, 0.04)	-0.23 (-0.92, 0.45)

*0.00 indicates a very small positive number < 0.005

**-0.00 indicates a very small negative number > -0.005

Table S4. Decadal trends in green-up and dormancy dates in different land cover classifications. Parentheses show the 95% credible intervals. Values are bolded if credible intervals do not overlap zero.

		Spring 10%	Spring 50%	Spring 90%	Fall 10%	Fall 50%	Fall 90%
Decadal trends in green-up/dormancy minus passage date (days/decade)	All pixels	**3.14 (0.77, 5.51)**	**3.51 (1.11, 5.91)**	**3.35 (0.93, 5.59)**	0.52 (-2.24, 3.24)	-0.62 (-3.32, 2.18)	-0.07 (-2.89, 2.71)
	Cropland	**2.44 (0.12, 4.54)**	**2.88 (0.61, 5.21)**	**2.59 (0.25, 4.98)**	0.19 (-2.55, 2.85)	-0.88 (-3.77, 1.93)	-0.51 (-3.17, 2.27)
	Grassy	**2.98 (0.39, 5.55)**	**3.36 (0.56, 6.05)**	**3.07 (0.38, 5.82)**	-0.41 (-3.6, 2.49)	-1.68 (-5.01, 1.46)	-1.07 (-4.16, 2.05)
	Woody	**3.67 (0.01, 7.42)**	**3.86 (0.2, 7.66)**	**3.82 (0.13, 7.58)**	0.8 (-1.53, 3.23)	-0.24 (-2.6, 2.1)	0.44 (-2.07, 2.86)
Decadal trends in green-up/dormancy date (days/decade)	All pixels		**2.67 (0.46, 4.84)**			1.05 (-1.45, 3.53)	
	Cropland		2.03 (-0.22, 4.2)			0.9 (-1.51, 3.12)	
	Grassy		**2.64 (0.18, 5.07)**			0.19 (-2.65, 2.99)	
	Woody		3.00 (-0.41, 6.56)			0.8 (-1.3, 2.97)	

Table S5. Decadal trends in green-up/dormancy and maturity/senescence. Parentheses show the 95% credible intervals. Values are bolded if credible intervals do not overlap zero.

	Spring 10%	Spring 50%	Spring 90%	Fall 10%	Fall 50%	Fall 90%
Decadal trends in green-up/dormancy minus passage date (days/decade)	**3.14 (0.77, 5.51)**	**3.50 (1.11, 5.91)**	**3.35 (0.93, 5.59)**	0.52 (-2.24, 3.24)	-0.62 (-3.32, 2.18)	-0.07 (-2.89, 2.71)
Decadal trends in maturity/senescence minus passage dates (day/decade)	**3.48 (0.96, 5.99)**	**3.84 (1.4, 6.42)**	**3.59 (1.00, 6.31)**	2.43 (-0.43, 5.14)	1.22 (-1.48, 3.94)	1.79 (-1.01, 4.49)
Decadal trends in green-up/dormancy date (days/decade)		**2.67 (0.46, 4.84)**			1.05 (-1.45, 3.53)	
Decadal trends in maturity/senescence date (days/decade)		**3.1 (0.69, 5.51)**			**3.39 (0.79, 5.98)**	
Trend in proximity to green-up/dormancy – Trend in green-up/dormancy date	-0.46 (-3.73, 2.83)	-0.46 (-3.73, 2.83)	-0.46 (-3.73, 2.83)	0.56 (-3.21, 4.26)	1.67 (-2.14, 5.4)	1.07 (-2.73, 4.79)
Trend in proximity to maturity/senescence – Trend in maturity/senescence date	-0.51 (-4.15, 3.02)	-0.51 (-4.15, 3.02)	-0.51 (-4.15, 3.02)	1.13 (-2.54, 4.9)	2.18 (-1.7, 5.92)	1.74 (-2.13, 5.47)

Table S6. Decadal trends in proximity of passage to green-up/dormancy date and in absolute green-up/dormancy date in two regions: west of 97°W and east of 97°W. The year 2012 was removed from the analysis because all residuals for this year were < 0.

Quantile	Land surface phenology – West		Land surface phenology – East	
	Proximity of passage to green-up (spring) or dormancy (fall) transition date	Absolute green-up (spring) or dormancy (fall) transition date	Proximity of passage to green-up (spring) or dormancy (fall) transition date	Absolute green-up (spring) or dormancy (fall) transition date
Spring 10%	**6.39 (1.78, 10.99)**	**5.60 (1.35, 9.79)**	1.42 (-0.17, 3.02)	**1.75 (0.5, 2.96)**
Spring 50%	**6.79 (2.57, 11.25)**		**2.1 (0.58, 3.6)**	
Spring 90%	**6.38 (1.96, 10.82)**		**2.24 (0.89, 3.64)**	
Fall 10%	3.43 (-1.85, 8.65)	3.74 (-0.62, 8.06)	0.73 (-0.99, 2.26)	3.14 (-1.48, 7.64)
Fall 50%	2.3 (-3, 7.49)		-0.25 (-1.73, 1.28)	
Fall 90%	1.71 (-3.65, 7.01)		1.12 (-0.3, 2.72)	

References

Ault TR, Henebry GM, de Beurs KM, et al (2013) The false spring of 2012, earliest in North American record. Eos 94:181-182. DOI:10.1002/2013EO20000

Section S3. Supplemental Tables and Figures

Figure S2 Coefficient estimates for decadal trends in environmental conditions on passage dates (black circles) and during passage periods (white triangles). The black circles represent the mean coefficient estimates for year, multiplied by ten, in models of change over time in environmental conditions on the dates when 10%, 50%, and 90% cumulative passage occurred each year. If changes to migration phenologies fully compensated for changes in environmental conditions (Figure 1d in main text), we expected these coefficients (black circles) to be near zero (dashed line). The white triangles represent the mean coefficient values for change in the environmental condition over time during the respective passage periods (passage periods defined in Figure 1b/c of main text). The shaded areas represent the posterior probability distributions (10%: pink, 50%: blue, 90%: orange), and the error bars represent the 95% credible intervals. For land surface phenology (LSP), the black circles represent the change in proximity to land surface phenophase transition date (green-up or dormancy date minus passage date) and the white triangles represent the change in absolute green-up or dormancy date.

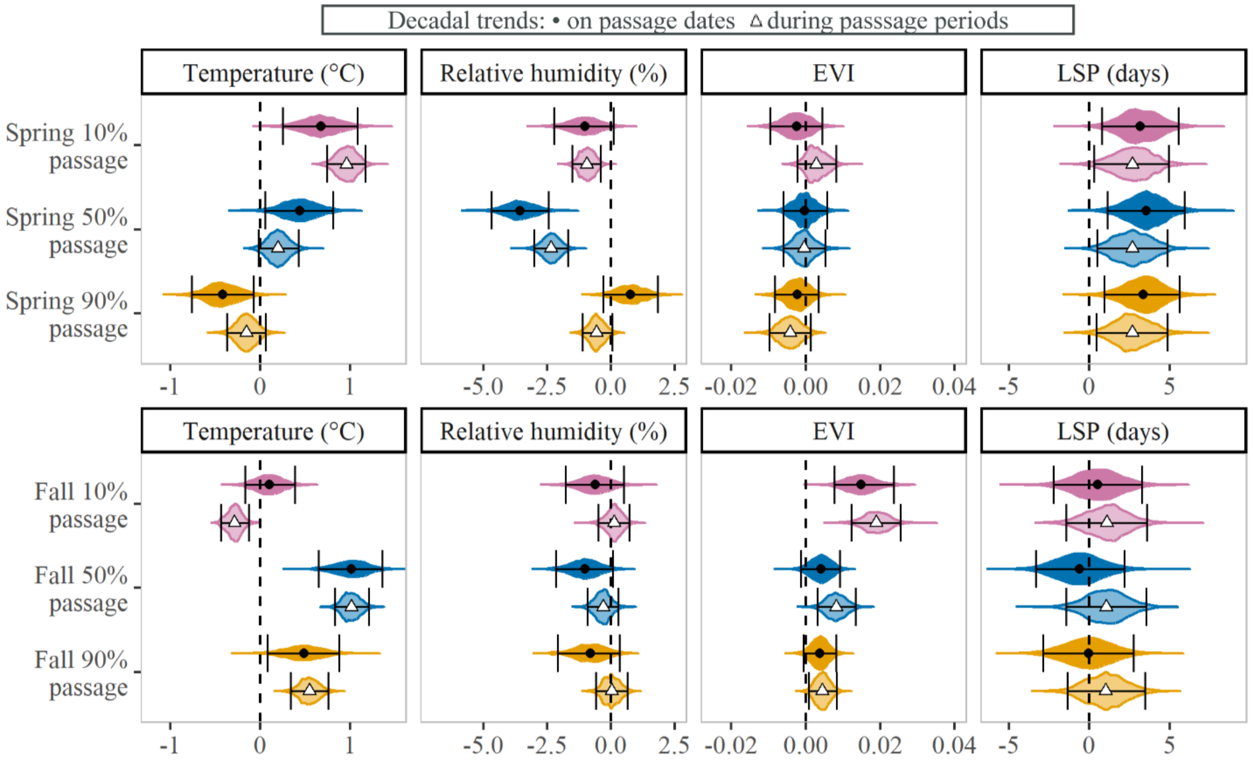

Table S7 Improvements in R^2 from including effects of long-term trends and interannual anomalies in environmental conditions on passage dates

Row number	Model	R^2: 1 – (sum of residuals squared/total sum of squares)					
		Spring 10%	Spring 50%	Spring 90%	Fall 10%	Fall 50%	Fall 90%
1	Random intercept for station + fixed effects of latitude, longitude, and latitude × longitude + random slope for the effect of year by station (null model)	0.65	0.79	0.69	0.62	0.75	0.85
2	+ fixed effects for interannual anomalies in climate oscillations	0.67	0.80	0.70	0.65	0.77	0.86
3	+ fixed effects for interannual anomalies in environmental conditions	0.77	0.83	0.72	0.67	0.80	0.88
4	R^2 for climate oscillation model – R^2 for null model	0.01	0.02	0.01	0.03	0.02	0.01
5	(R^2 for climate oscillation model) / (1 - R^2 for null model)<br><br>Proportion of remaining variance explained by adding the climate oscillation modes	0.04	0.07	0.03	0.08	0.09	0.06
6	R^2 for full model – R^2 for climate oscillation model	0.10	0.03	0.02	0.02	0.03	0.02
7	(R^2 for full model – R^2 for climate oscillation model) / (1 - R^2 for climate oscillation model)<br><br>Proportion of remaining variance explained by adding the interannual anomalies in environmental conditions	0.31	0.13	0.08	0.05	0.12	0.14

We calculated the total sum of squares (SST) as the sum of the squared distance of each passage date from the mean passage date. Our models generated posterior probability distributions for the residuals for each data point (observed value – expected value based on model), and we summed the squares of the means of these posterior distributions to calculate the sum of squared residuals (SSR). We calculated R^2 as 1 – SSR/SST
